# High Glucose Induces Late Differentiation and Death of Human Oral Keratinocytes

**DOI:** 10.3390/cimb44090275

**Published:** 2022-09-04

**Authors:** Junhe Shi, Chen Han, Dandan Chen, Harsh M. Trivedi, Hiba I. Bangash, Lin Chen

**Affiliations:** 1 Institute of Clinical Pharmacology, Xiyuan Hospital, China Academy of Chinese Medical Sciences, Beijing 100091, China; 2 Department of Periodontics, College of Dentistry, University of Illinois Chicago, Chicago, IL 60612, USA; 3 Center for Wound Healing and Tissue Regeneration, University of Illinois Chicago, Chicago, IL 60612, USA; 4 Colgate-Palmolive Company, Piscataway, NJ 08854, USA

**Keywords:** high glucose, gingiva, keratinocyte, differentiation, apoptosis, necrosis

## Abstract

Keratinocytes are essential cells for wound repair. Impaired oral wound healing is common in diabetic patients with periodontal disease. High glucose, or hyperglycemia, impairs the cellular function of different cell types. However, it is unknown whether high glucose has a detrimental effect on the functions of oral keratinocytes. In the current study, a human gingival keratinocyte cell line, telomerase immortalized gingival keratinocytes (TIGK), was treated with high glucose (24 and 48 mM) for up to 120 h. Proliferation, migration, cell viability, and production of markers of differentiation, growth factors and enzymatic antioxidants were assessed after high glucose treatment. The results showed that high glucose significantly inhibited TIGK proliferation and migration. High glucose also induced significant cell death through apoptosis and necrosis as determined by flow cytometry, especially at 120 h after high glucose treatment. Necrosis was the dominant form of cell death induced. Real-time PCR showed that high glucose treatment upregulated mRNA expression of late keratinocyte differentiation makers, such as keratin 1, 10, 13 and loricrin, and downregulated enzymatic antioxidants, including superoxide dismutase 1, catalase, nuclear factor erythroid 2 -related factor 2, heme oxygenase 1. In conclusion, high glucose impairs the proliferation and migration of oral keratinocytes and likely induces cell death through the promotion of late cell differentiation and down-regulation of enzymatic antioxidants.

## 1. Introduction 

The oral epithelium is either keratinized or nonkeratinized. Different layers of epithelia can be identified as the basal layer, spinous layer, granular layer for keratinized epithelia, or intermediate layer (stratum intermedium) for nonkeratinized epithelia [1,2,3,4]. Oral epithelia undergo constant renewal or differentiation from the lower layers to the upper layers. The epithelia contain a few other cell types besides keratinocytes, including melanocytes, Langerhan cells, Merkel cells, and lymphocytes [1,2,3,4]. However, keratinocytes are the major cell population in the epithelia. Re-epithelization through keratinocyte proliferation and migration is a hallmark of wound repair and results in layers of tightly connected keratinocytes that serve as a strong physical barrier [5,6]. Following tissue injury, keratinocytes can also be activated to produce growth factors such as FGFs, VEGF, TGFβ, and EGF, numerous cytokines and chemokines [7,8], and anti-microbial peptides [9,10]. These factors are pivotal for wound healing as they play critical roles in re-epithelization, fibroblast proliferation, angiogenesis, and the immune response [5,6]. 

Diabetes is not only closely associated with chronic skin wounds or ulcers, but also with impaired oral mucosal wound healing [11,12,13,14]. Impaired oral wound healing in diabetic rats shows a more severe inflammatory response [11]. Delayed cheek mucosal wound closure in diabetic rats [12] is related to a prolonged inflammation that is marked by overexpression of TNF-α and IL-1β, decreased FGF-2 expression, impaired angiogenesis, and decreased collagen deposition [12]. Additionally, impaired wound healing following tooth extraction in diabetic mice has been linked to decreased TGFβ expression [13,14]. In regard to the specific cell types affected by diabetes during oral wound healing, multiple studies have already assessed the effects of diabetes and hyperglycemia on fibroblasts. One study showed that impaired oral wound healing in diabetic mice was associated with decreased proliferation and increased apoptosis of fibroblasts [15]. Another study found that high glucose concentrations could induce oxidative stress in human gingival fibroblasts in vitro and, subsequently, impair gingival fibroblast proliferation and migration [16]. Taken together, these studies suggest that fibroblast malfunction may be a contributor to impaired oral wound healing in diabetes. In general, factors that lead to poor healing are related to degrees of high glucose levels, elevated inflammation, impaired angiogenesis, increased levels of advanced glycation end products, and excessive formation of reactive oxygen species (ROS) [12,17,18,19,20,21]. In a broader sense, these studies provide evidence to suggest that diabetes, or more specifically, hyperglycemia, can induce cellular dysfunction that may contribute to impaired wound healing. Therefore, an investigation into the effects of hyperglycemia on other cell types vital to wound healing is necessary to better understand the healing deficits seen in diabetic patients. One such cell type is the keratinocytes, which are critical for re-epithelialization during wound repair. Though many studies have demonstrated that a high glucose environment decreases skin keratinocyte migration and proliferation in vitro [22,23,24,25], the effects of high glucose conditions on oral keratinocytes have not been explored. To the best of our knowledge, the current investigation is the first study to answer this question. The results from this investigation will help us understand other mechanisms that lead to impaired oral wound healing in diabetes.

## 2. Materials and Methods

### 2.1. Cell Culture

Telomerase Immortalized Gingival Keratinocytes/TIGK were purchased from ATCC (Manassas, VA, USA). TIGK cells were cultured in a commercially available serum-free culture medium (Lifeline Cell Technology, Frederick, MD, USA) containing insulin (5 µg/mL), L-Glutamine (6 mM), epinephrine (1 µM), apo-transferrin (5 µg/mL), and TGF-α (0.5 ng/mL), pituitary extract (0.4%), and hydrocortisone hemisuccinate (100 ng/mL). D-glucose concentration in this medium was 6 mM, which was considered low glucose. For higher glucose concentrations, 24 and 48 mM were used, and extra D-Glucose (Sigma-Aldrich, St. Louis, MO, USA) was added to the medium during the testing.

### 2.2. Proliferation Assay

TIGK cells were cultured in a T75 flask in serum-free medium at 37 °C with 5% CO_2_. When cells reached 80% confluency, TrypLE (Thermo Fisher Scientific, Waltham, MA, USA) was used to detach the cells. A total of 8000 cells (for 24 and 48-h proliferation) and 5000 cells (for 72 and 120-h proliferation) were seeded in each well of a 96-well plate. Twelve hours later, the media was switched to ones with high concentrations of glucose (24 mM and 48 mM). Cell proliferation was examined using an MTS Cell Proliferation Assay Kit (Abcam, Cambridge, UK) 24, 72, and 120 h after glucose treatment according to the instructions on the kit. Optical density (OD) at 490 (OD_490_) was recorded with a spectrophotometer (Molecular Devices, San Jose, CA, USA). Results were shown as the percent of OD_490_ values of the low glucose level at 6 mM at each time point. There were 3–6 replicates for each culture condition and time point. 

### 2.3. Migration Assay

TIGK cells were seeded in a 3-well silicone insert (ibidi, Fitchburg, WI, USA) in a well of a 12-well plate. Silicon culture inserts were used to create gaps with a width of 500 µm. Each well was planted with 50,000 cells in 70 μL of the medium. Each 3-well insert created two gaps. When cells reached 100% confluency, they were treated with 1µg/mL mitomycin C (Sigma-Aldrich St. Louis, MO, USA) for 1 h to stop cell proliferation and then washed 3 times with PBS. After the inserts were removed, the culture media was switched to ones with higher concentrations of glucose (24 mM and 48 mM). Images of the gaps were taken at 0 h, 12 h, 24 h, and 48 h post-gap removal. The gap area in pixels was assessed by ImageJ software. Each treatment at each time point had 4 replicates. The percentage of the opened area was calculated based on the initial gap area. There were 3–6 replicates for each culture condition and each time point. 

### 2.4. Real-Time PCR

TIGK cells were cultured in 6-well plates and then seeded with 3 × 10^5^ cells/well. When cells reached 50% confluency (for 24 and 72 h-samples) or 30% confluency (for 120 h samples), the culture media was switched to ones with higher concentrations of glucose (24 mM and 48 mM) in triplicates. After 24, 72 and 120 h of treatment, the cells were harvested with TriZol (Invitrogen Waltham, MA, USA), and the total RNA was then extracted according to the instructions of the manufacturer. A total of 1µg of total RNA was then treated with DNAse I (Invitrogen, Waltham, MA, USA) and converted to cDNA using a High-Capacity cDNA Reverse Transcription Kit (Thermo Fisher Scientific, Waltham, MA, USA). Quantitative real-time PCR was carried out using a StepOne Plus real-time PCR system (Applied Biosystems, Waltham, MA, USA) with the SYBR Green mix (Roche, Basel, Switzerland) and specific primers. The targets were keratinocyte differential markers including loricrin, keratin 1 (KRT1), keratin 10 (KRT10), and keratin 13 (KRT13), growth factors including KGF1 (FGF7) and KGF2 (FGF10), antioxidants including superoxide dismutase 1 (SOD1), catalase (CAT), nuclear factor erythroid 2 -related factor 2 (NRF2), heme oxygenase 1 (HO1), and late apoptosis pathway molecules including caspase 3 (Casp3) and 7 (Casp7), and necrosis marker of receptor-interacting serine/threonine-protein kinase 3 (RIPK3). Primer sequences are listed in Table 1. Relative expression was calculated using the 2^−∆∆CT^ method. Glyceraldehyde 3-phosphate dehydrogenase (GAPDH) was used as the housekeeping gene for calibration. The expression of the targets in the cells with low glucose control (6 mM) treatment at each time point was used as the baseline to calculate the relative expression of high glucose-treated samples. There were 3 replicates for each culture condition and time point. 

### 2.5. Flow Cytometry

TIGK cells were cultured and treated in the same conditions described in the Real-time PCR section. 

TIGK cells treated with different concentrations of glucose, including both attached and disassociated, were harvested. Early apoptotic, late apoptotic, necrotic, and live cells were determined using an Annexin V Apoptosis Detection Kit APC (Thermo Fisher Scientific, Waltham, MA, USA). Harvested cells were washed with binding buffer. After centrifugation, cells were re-suspended in 100 μL of binding buffer and incubated with APC-conjugated Annexin V for 15 min at room temperature. After washing, 5 μL of propidium iodide (PI) was added. The cells were then immediately subjected to flow cytometry analysis without being fixed by any fixatives using LSR Fortessa (BD bioscience, San Jose, CA, USA) at the Flow Cytometry Core, Research Resources Center, University of Illinois Chicago. Results were analyzed using Summit 4.3 software (Dako Cytomation, Glostrup, Denmark). Cell debris and doublets were gated out in the analysis. There were 3 replicates at each culture condition and each time point.

### 2.6. Statistical Analysis

All values were expressed as mean + SD. One-way or two-way ANOVA followed by a post hoc test was used for statistical analysis (GraphPad Software Inc., San Diego, CA, USA). Statistical significance was defined at a *p*-value < 0.05. 

## 3. Results

### 3.1. High Glucose Impedes the Proliferation of Oral Keratinocytes 

**Figure 1:**
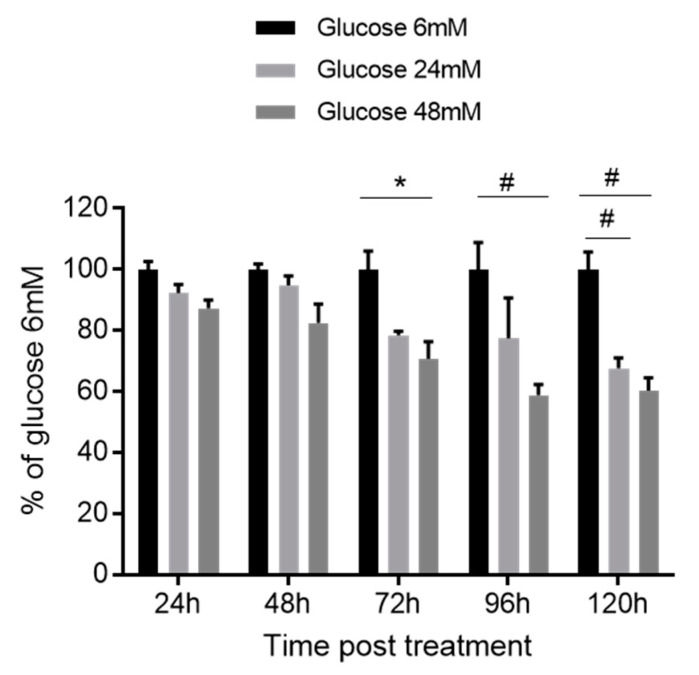
**High glucose inhibits the proliferation of oral keratinocytes.** TIGK cells were seeded in a 96-well plate. Twelve hours later, the media was switched to ones with higher concentrations of glucose (24 mM and 48 mM). Cell proliferation was examined using an MTS assay 24, 72, and 120 h after the treatment. Results are shown as the percent of OD_490_ values of the 6 mM glucose treatment at each time point. There were 6 replicates in the low glucose group and 3 replicates in each high glucose group for each time point. * *p* < 0.05, # *p* < 0.01. One-way ANOVA followed by Tukey’s post-tests was used for statistical analysis.

TIGK cells were cultured under high glucose concentrations of 24 mM or 48 mM for 24–120 h. Proliferation rates of TIGK cells cultured under a high glucose concentration of 24 mM were significantly lower compared to the lower glucose concentration of 6 mM only 120 h after the treatment (Figure 1, *p* < 0.01). However, proliferation rates under a high glucose concentration of 48 mM were significantly lower compared to the lower glucose concentration 72, 96, and 120 h after the treatment (Figure 1, *p* < 0.05–0.01). There seemed to be a time and dose-dependent inhibitory effect, but no statistical significance was observed between glucose concentrations of 24 mM and 48 mM for any time point (Figure 1, *p* > 0.05). 

### 3.2. High Glucose Inhibits Migration of Oral Keratinocytes 

**Figure 2:**
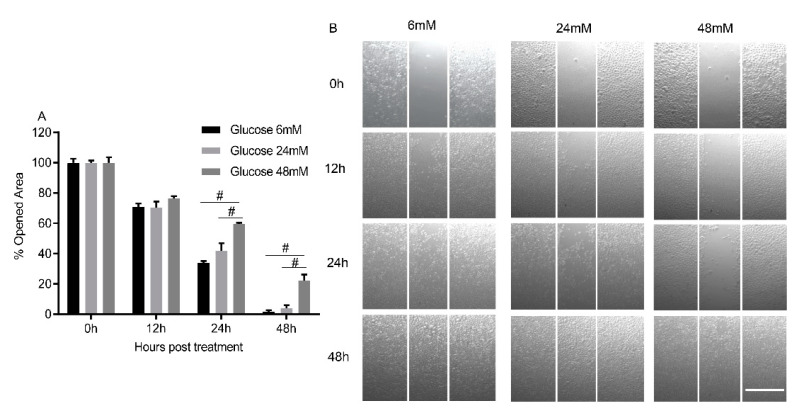
**High glucose inhibits the migration of oral keratinocytes.** TIGK cells were seeded in a 3-well silicone insert. When cells reached 100% confluence, cells were treated with 1µg/mL mitomycin C to stop cell proliferation. After the inserts were removed, culture media was switched to ones with higher concentrations of glucose (24 mM and 48 mM). Images were taken at 0, 12, 24, and 48 h. Gap area in pixels was assessed by ImageJ software. There were 6 replicates in the low glucose group (6 mM) and 3 replicates in each high glucose group for each time point. Percent of opened areas was calculated based on the initial gap area. (**A**). Summary of TIGK cell migration under high glucose conditions. # *p* < 0.01. One-way ANOVA followed by Tukey’s post-test was used for statistical analysis. (**B**). Representative images of TIGK cell migration. Scale bar: 500µM.

The high glucose concentration of 48 mM significantly inhibited TIGK migration at 24 and 48 h after treatment as compared to the low glucose concentration of 6 mM (Figure 2A,B, *p* < 0.01). The glucose concentration of 24 mM seemed to inhibit TIGK migration at 24 and 48 h compared to the low glucose concentration of 6 mM but was not statistically significant (*p* > 0.05). 

### 3.3. High Glucose Induces Oral Keratinocyte Cell Death 

**Figure 3:**
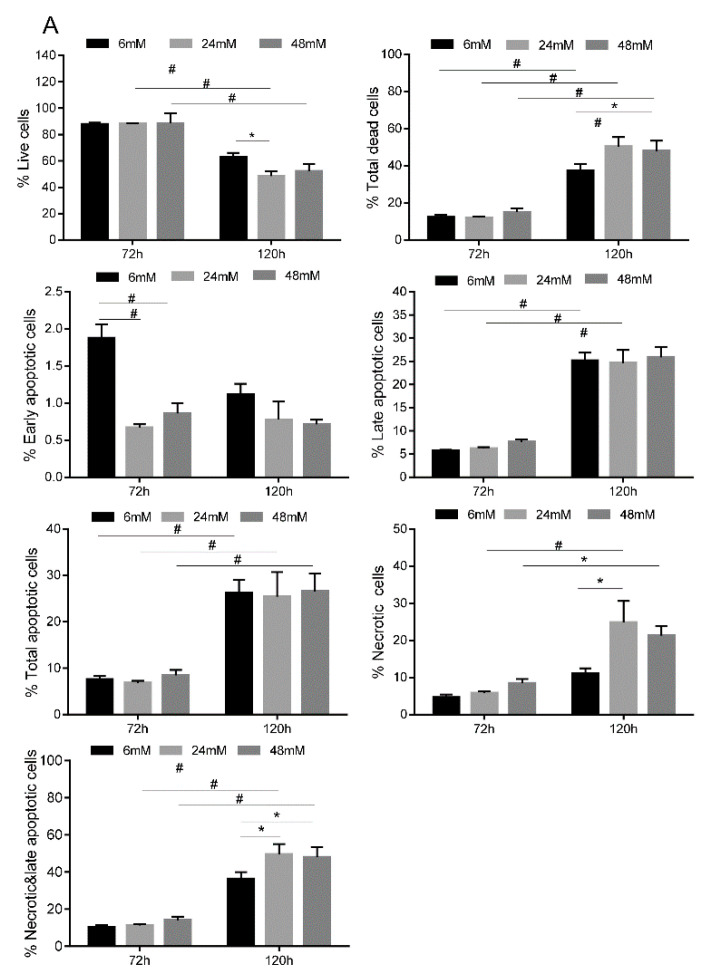

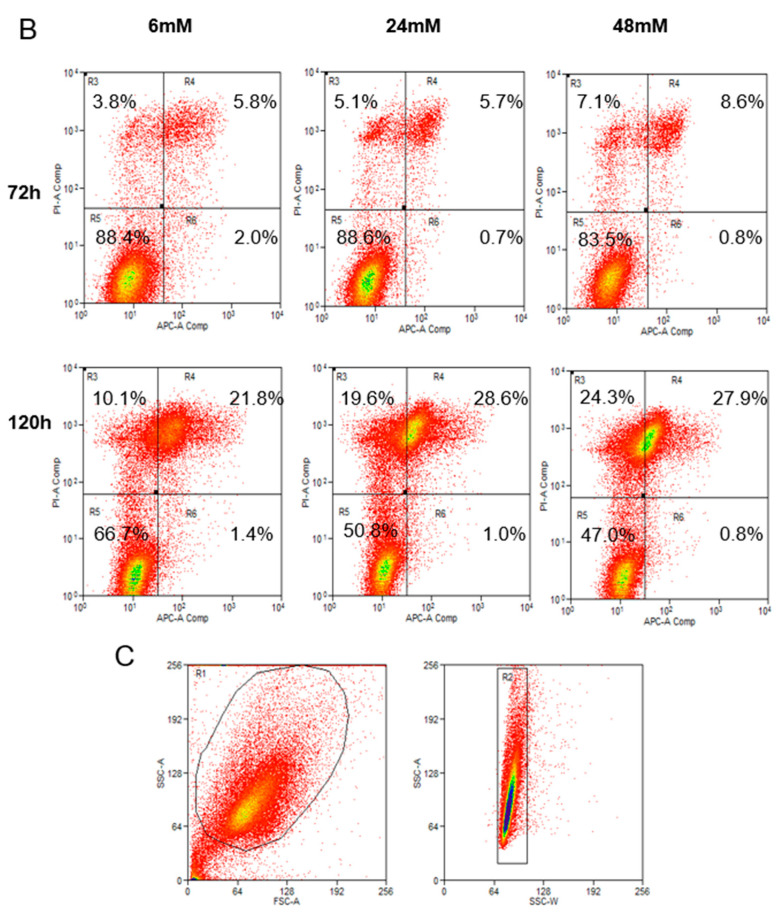
**High glucose induces oral keratinocyte cell death.** TIGK cells treated with different concentrations of glucose were harvested, stained with an Annexin V Apoptosis Detection Kit APC, and analyzed by flow cytometry. (**A**) Summary of TIGK cell viability and death under high glucose conditions. There were 3 replicates for each culture condition and time point. * *p* < 0.05, # *p* < 0.01. (**B**) Representative images of flow cytometry analysis. R3: necrotic cells, R4: late apoptotic cells, R5: live cells, R6: early apoptotic cells. Note: the results (percentages) described in the result section are from Figure 3A and are the averages of 3 replicates. Therefore, they are slightly different from the representatives shown in Figure 3B, which are based on 1 of the 3 replicates. (**C**). Flow cytometry gating strategies. Cell debris and doublets were gated out in the analysis. Two-way ANOVA followed by Bonferroni’s post-tests was used.

TIGK cells were cultured under glucose concentrations of 6 mM, 24 mM or 48 mM for 72 and 120 h. Live cells, apoptotic, and necrotic cells were identified using an Annexin V Apoptosis Detection Kit as described in the Materials and Methods section. There were similar live cell accounts (R5, PI and Annexin V negative cells), about 88%, at 72 h for the different glucose concentrations of 6 mM, 24 mM or 48 mM (Figure 3A,B). However, at 120 h, there was a significantly lower percentage of live TIGK cells for the 24 mM high glucose treatment group (48.4 ± 3.7%, *p* < 0.05) as compared to the 6 mM low glucose control (62.7 ± 3.5%) (Figure 3A,B). The percentage of live TIGK cells for the 48 mM high glucose treatment group (52.1 + 5.6%) was similarly lower than the 6 mM low glucose control 99 (62.7 + 3.5%) but was not statistically significant (*p* > 0.05, Figure 3A,B). At 72 h, there was no difference in the total percent of dead cells observed among the different glucose concentrations (Figure 3A,B). At 120 h, the percent of total dead TIGK cells (R3 necrotic cells + R4 late apoptotic cells + R6 early apoptotic cells) was 50.3 + 5.2% (*p* < 0.01) at 24 mM and 47.9 + 5.6% (*p* < 0.05) at 48 mM high glucose conditions, respectively, which were significantly higher than 37.3 + 3.5% at 6 mM low glucose control (Figure 3A,B).

**Figure 4:**
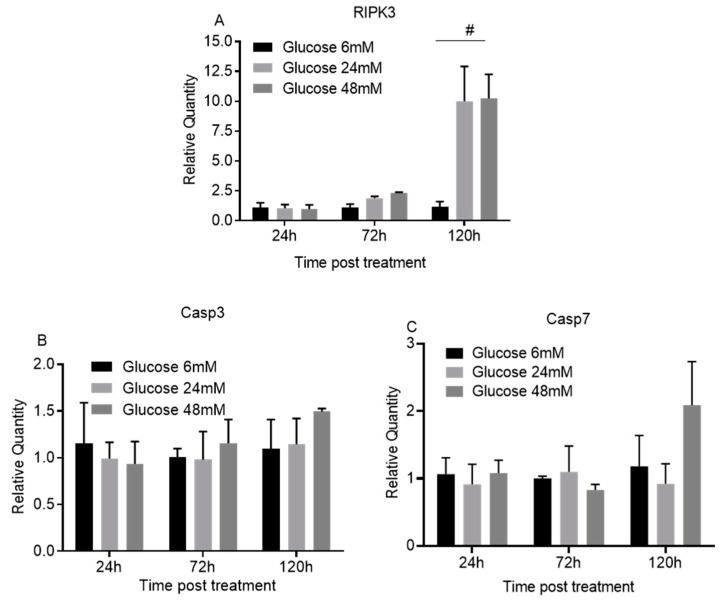
**High glucose upregulates necrosis but not late apoptosis related genes in oral keratinocytes.** TIGK cells treated with different concentrations of glucose (6, 24, and 48 mM) were harvested. Total RNA was extracted and cDNA was prepared. Real time PCR was performed to detect the mRNA expression of RIPK3 (**A**), Caspase 3 (**B**), and Caspase 7 (**C**). There were 3 replicates for each culture condition and time point. The expression of the targets in the cells with 6 mM glucose treatment at each time point was used as the baseline. # *p* < 0.01. One-way ANOVA followed by Tukey’s post-tests was used for statistical analysis.

Small numbers of early apoptotic cells (R6) at both 72 and 120 h were identified. Interestingly, at 72 h, there was a significantly lower percent of early apoptotic cells for the 24 mM (0.7 ± 0.1%, *p* < 0.01) and 48 mM high glucose treatment groups (0.9 ± 0.2%, *p* < 0.01) as compared to the 6 mM low glucose control (1.9 ± 0.3%). However, no significant difference in the percent of early apoptotic cells was observed among the different glucose concentrations at 120 h (Figure 3A,B). There was a significantly higher percent of late apoptotic cells (R4) at 120 h compared to 72 h for all three glucose concentrations: 6 mM (25.1 + 3.0 vs. 5.7 ± 0.3, *p* < 0.01), 24 mM (24.6 ± 4.9 vs. 6.2 ± 0.5, *p* < 0.01) and 48 mM (25.8 ± 3.8% vs. 7.6 ± 1.1%, *p* < 0.01). There was no significant difference in late apoptotic cells between low and high glucose groups at either time point (Figure 3A,B). Similar results were found for total apoptotic cells (R4 + R6) (Figure 3A,B). However, there were significantly more necrotic cells (R3), especially at 120 h, for the 24 mM (24.9 ± 10.0, *p* < 0.05) and 48 mM (21.3 ± 2.6%, *p* < 0.05) high glucose concentrations as compared to the 6 mM low glucose concentration (11.1 ± 1.4%) (Figure 3A,B). Similarly, there were significantly more total necrotic plus late apoptotic cells (R3 + R4) at 120 h for the high glucose groups (Figure 3A,B) than for the low glucose control. This suggests that high glucose induces cell death predominately through necrosis, not apoptosis. This is further supported by how treatment with either the 24 mM or 48 mM high glucose concentration for 120 h significantly upregulated RIPK3 (*p* < 0.01, Figure 4A), a cell necrosis-related gene, and not downstream apoptosis-inducing genes such as caspase 3 and 7 (*p* > 0.05, Figure 4B,C). 

### 3.4. High Glucose Upregulates Expression of Late Differentiation Markers in Oral Keratinocytes 

**Figure 5:**
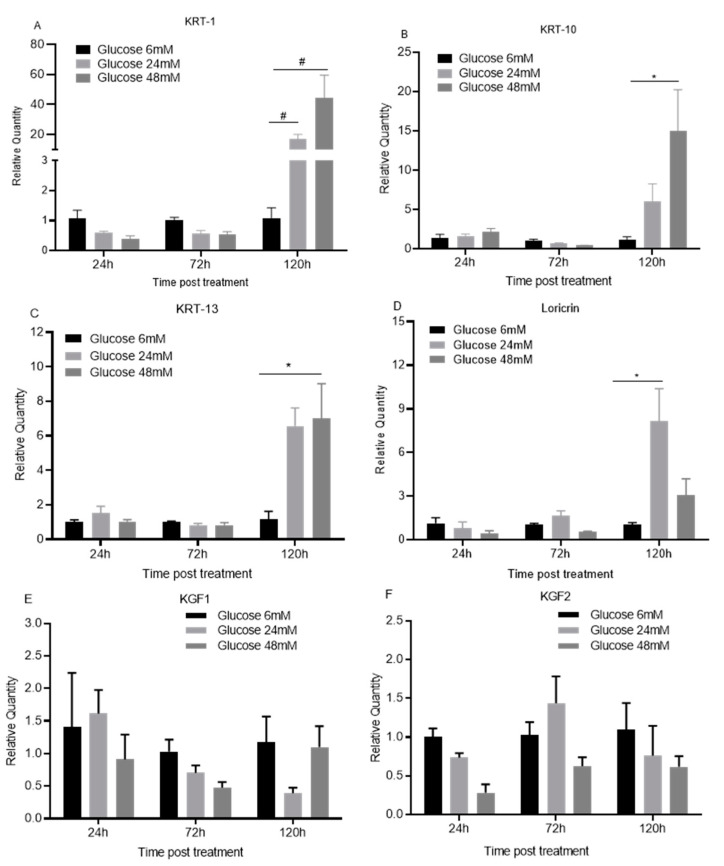
**High glucose upregulates late differentiation markers in oral keratinocytes.** TIGK cells treated with different concentrations of glucose (6, 24, 48 mM) were harvested. Total RNAs were extracted and cDNAs were prepared. Real-time PCR was performed to detect the mRNA expression of KRT-1 (**A**), KRT-10 (**B**), KRT-13 (**C**), loricrin (**D**), KGF1 (**E**) and KGF2 (**F**). There were 3 replicates at each culture condition and each time point. The expression of the targets in the cells with 6 mM treatment at each time point was used as the baseline. * *p* < 0.05, # *p* < 0.01. One-way ANOVA followed by Tukey’s post-tests was used for statistical analysis.

120 h after treatment with high glucose concentrations of 24 or 48 mM, mRNA expression of late keratinocyte differentiation markers, including KRT-1, KRT10, KRT-13, and loricrin, were significantly enhanced when compared to treatment with low glucose concentrations of 6 mM (*p* < 0.05–0.01, Figure 5A–D). However, high glucose treatment did not significantly affect the expression of these molecules at 24 or 72 h (*p* > 0.05, Figure 5A–D). The results indicate that high glucose promotes TIGK cells to differentiate towards a late or terminal stage of the cells.

High glucose concentrations of 24 and 48 mM seemed to also have a dose-dependent inhibition of mRNA expression of growth factors, KGF1 and KGF2, from 24 to 120 h after the treatment compared to those treated with low glucose. However, there was no statistical significance (*p* > 0.05, Figure 5E,F).

### 3.5. High Glucose Downregulates the Expression of Enzymatic Antioxidants

**Figure 6:**
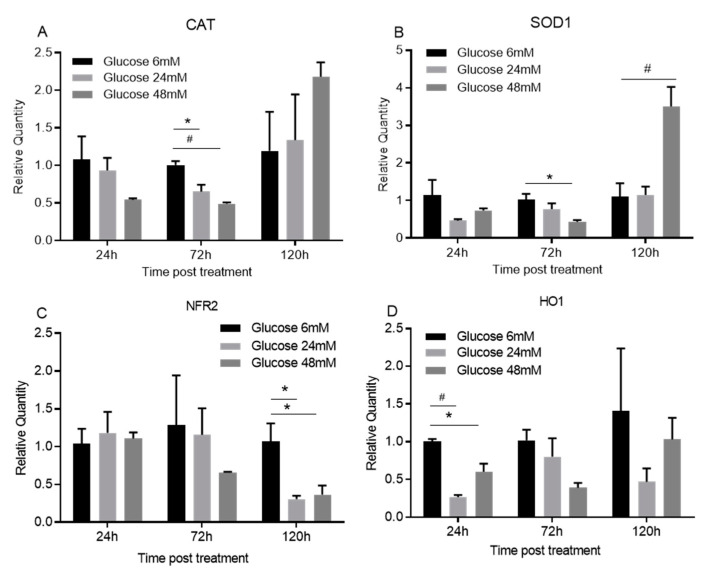
**High glucose inhibits the expression of antioxidant enzymes in oral keratinocytes.** TIGK cells treated with different concentrations of glucose (6, 24, 48 mM) were harvested. Total RNAs were extracted and cDNAs were prepared. Real time PCR was performed to detect the mRNA expression of CAT, SOD1, NFR2, and HO1. There were 3 replicates at each culture condition. The expression of the target genes in cells treated with 6 mM glucose at each time point was used as the baseline. * *p* < 0.05, # *p* < 0.01. One-way ANOVA followed by Tukey’s post-tests was used for statistical analysis.

Treatment with high glucose concentrations significantly downregulated the mRNA expression of antioxidants CAT, SOD1, NFR2, and HO1 when compared to treatment with a low glucose concentration of 6 mM (Figure 6A–D). High glucose concentrations of 24 and 48 mM decreased the expression of CAT at 72 h (Figure 6A, *p* < 0.05 and 0.01). The high glucose concentration of 48 mM decreased the expression of SOD1 at 72 h (Figure 6B, *p* < 0.05), while high glucose concentrations of 24 and 48 mM decreased the expression of NFR2 at 120 h (Figure 6C, *p* < 0.05). Additionally, high glucose concentrations of 24 mM and 48 mM both downregulated the expression of HO1 at 24(Figure 6D, *p* < 0.05 and 0.01). Interestingly, the high glucose concentration of 48 mM significantly upregulated SOD1 expression 120 h after the treatment (Figure 6B, *p* < 0.01).

## 4. Discussion

Our study demonstrates that high glucose conditions significantly inhibit the proliferation and migration of human oral keratinocytes, which may partially explain why oral wound closure is delayed in diabetes. This observation is consistent with previous studies analyzing the effects of hyperglycemia on human skin keratinocytes [22,23,24,25]. Furthermore, high glucose levels induced oral keratinocyte cell death. The longer the cells were subject to high glucose culture conditions, the more dead cells there were when compared to a low glucose culture condition. We observed that there were two dead cell populations among cells cultured in either low glucose or high glucose conditions: apoptotic cells and necrotic cells. There was no difference in the proportion of apoptotic cells between the low glucose and high glucose conditions. However, there were significantly more necrotic cells than apoptotic cells for oral keratinocytes cultured in high glucose conditions. Therefore, high glucose likely induces cell death through necrosis and not apoptosis. This was further supported by significantly increased RIPK3 expression, an indicator of necrotic cell death [26,27]. Additionally, there were no significant changes in the expression of caspase 3 and caspase 7, indicators of downstream apoptosis cascade activation, between cells cultured in low or high glucose conditions, which is consistent with a previous publication [28]. 

We next investigated whether high glucose treatment would affect oral keratinocyte differentiation. The mRNA expression of KRT1, KRT 10, KRT 13, and loricrin was found to be significantly increased under high glucose conditions. These molecules are late differentiation markers of keratinocytes [29,30,31,32]. Similar results for KRT10 and loricrin expression were observed in the skin of type I diabetic mice [28]. Altogether, our results suggests that high glucose may promote oral keratinocytes to differentiate into a later or more terminal cell stage. 

Moreover, we found that high glucose seemed to inhibit the expression of growth factors KGF 1 and KGF 2 in oral keratinocytes even though it was not statistically significant. KGF1 (FGF7) and KGF2 (FGF10) belong to the family of fibroblast growth factors. KGFs induce keratinocyte proliferation and migration [33,34] and are significantly increased in acutely injured skin of both mice and humans [34,35]. In addition, reduced KGF expression during wound repair was observed in healing-impaired diabetic animals [36]. These observations suggest that KGFs are important growth factors, especially in diabetic wound repair. 

It is well known that excessive production of ROS is partially responsible for diabetic ulcers or chronic wounds [17,18,37]. In our study, we found that high glucose inhibited the expression of enzymatic antioxidants including CAT, SOD1, NFR2, and HO1, which confirmed previously published studies [16,37,38,39,40], suggesting that enzymatic antioxidants were downregulated and there was a probable excessive production of ROS induced by high glucose conditions.

Taken together, our major findings in the current study are that high glucose significantly impairs the proliferation and migration of oral keratinocytes. High glucose induces the expression of late differentiation markers and necrosis of oral keratinocytes. High glucose impairs the expression of enzymatic antioxidants. Understanding the detrimental effects of high glucose on oral keratinocytes and its underlying mechanism will help us develop strategies to treat impaired diabetic oral wound healing.

Limitations of the current study and future directions: The current study demonstrated that upregulated RIPK3 expression might be involved in high glucose-induced necrosis. However, how RIPK3 and its signaling pathway participate in the process needs to be further studied in oral keratinocytes. The study also observed that enzymatic antioxidants were inhibited by high glucose in oral keratinocytes. However, we did not know if ROS production was indeed significantly increased. Studies measuring ROS production under high glucose conditions will determine if high glucose conditions can induce excessive production of ROS. If high glucose conditions do induce excess ROS production, experiments measuring cell proliferation and migration after blocking ROS production under high glucose conditions will be necessary to determine if increased ROS production is responsible for the observed decrease in cell proliferation and migration. The study was based on an immortalized gingival keratinocyte cell line. Replication of our results in primary oral keratinocyte cells will strengthen the findings. Finally, the study lacks direct evidence showing that the observed phenomena in *in vitro* cell culture following the manipulation of extracellular glucose concentrations also occur in vivo in diabetic conditions. Thus, investigation of patient samples or experiments involving animal models is warranted.

## Figures and Tables

**Table 1:** Primer sequences for real-time PCR.

Targets	Forward (5′—3′)	Reverse (5′—3′)
GAPDH	CAGGGCTGCTTTTAACTCTGG	TGGGTGGAATCATATTGGAACA
loricrin	CGAAGGAGTTGGAGGTGTTT	GGCTTCTTCCAGGTAGGTTAAG
KRT1	ATCAATCTCGGTTGGATTCG	TCCTGCTGCAAGTTGTCAAG
KRT10	GCTGACCTGGAGATGCAAAT	AGCATCTTTGCGGTTTTGTT
KRT13	CAGCAGATCCAGGGACTCAT	TCTGGCACTCCATCTCACTG
KGF1 (FGF7)	CGTGCTTCCACCTCGTCT	TCTCCTGGGTCCTTTCA
KGF2 (FGF10)	GAGGCTGCAGTGAGCTATAATC	CCTTCCTTCCTTCCTGTCTTTC
SOD1	CCAGTGCAGGGCATCATCAA	TCTTCATCCTTTGGCCCACC
CAT	CGGACATGGTCTGGGACTTC	AACTGCCTCCCCATTTGCAT
HO1	ACATCCAGCTCTTTGAGGAGT	TGAGTGTAAGGACCCATCGGA
NFR2	TTCTCCCAATTCAGCCAGCC	AACGTAGCCGAAGAAACCTCA
Caspase 3	ACT GGA CTG TGG CAT TGA	GAG CCA TCC TTT GAA TTT CGC
Caspase 7	TGG TAG CAG TGG GAT TTG TG	CTG AAG AGG GAC GGT ACA AAC
RIPK3	CATAGGAAGTGGGGCTACGAT	AATTCGTTATCCAGACTTGCCAT

## Data Availability

Data are available upon request to the Corresponding Authors.
